# A new toolkit to visualize and perturb endogenous LIN-12/Notch signaling in *C. elegans*

**DOI:** 10.17912/micropub.biology.000603

**Published:** 2022-07-28

**Authors:** Ariel M Pani, Theresa V Gibney, Taylor N Medwig-Kinney, David Q Matus, Bob Goldstein

**Affiliations:** 1 Department of Biology, University of Virginia, Charlottesville, VA, USA; 2 Department of Cell Biology, University of Virginia School of Medicine, Charlottesville, VA, USA; 3 Department of Biochemistry & Cell Biology, Stony Brook University, Stony Brook, NY, USA; 4 D.Q.M. is a paid consultant of Arcadia Science; 5 Department of Biology, University of North Carolina at Chapel Hill, Chapel Hill, NC, USA; 6 Lineberger Comprehensive Cancer Center, University of North Carolina at Chapel Hill, Chapel Hill, NC, USA

## Abstract

Notch signaling mediates cell-cell interactions during development and homeostasis. Methods for visualizing and manipulating Notch activity *in vivo* are essential to elucidate how the Notch pathway functions. Here, we provide new tools for use in *C. elegans* to visualize and perturb Notch signaling *in vivo* using endogenously tagged alleles of the Notch receptor *lin-12* . Tagging the endogenous LIN-12 intracellular domain with the fluorescent protein mNeonGreen (mNG) allowed for visualization of both its membrane-localized state and translocation of the Notch intracellular domain into the nucleus upon ligand activation. LIN-12::mNG localized to the nucleus in cells where and when Notch signaling is known to be active and provided a real-time readout of Notch activity *in vivo* that complements existing biosensors and transcriptional reporters. We also report an allele of endogenous *lin-12* that we tagged with both mNG and an auxin-inducible degron, to facilitate conditional LIN-12 protein degradation. This toolkit provides novel reagents for the *C. elegans* research community to investigate mechanisms of Notch signaling and its functions *in vivo* .

**Figure 1. Endogenously tagged LIN-12 alleles are tools to visualize and manipulate native LIN-12/Notch activity in vivo.**
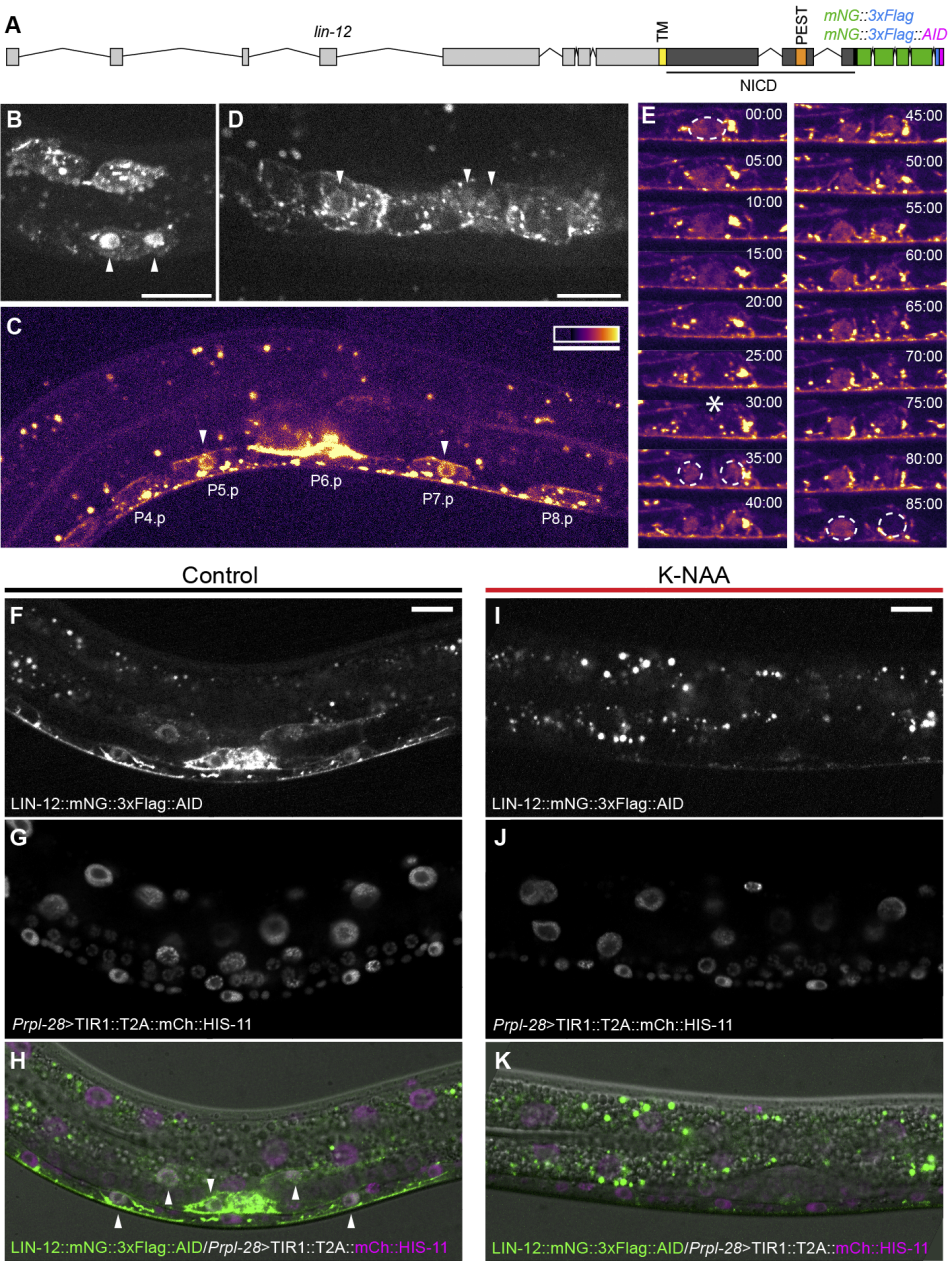
**A** , schematic of the endogenous *lin-12* locus in *lin-12::mNG::3xFlag* and *lin-12::mNG::3xFlag::AID* strains; The intracellular domain of LIN-12 is represented in dark gray with the transmembrane (TM) and PEST domains annotated. *mNG* is fused to the C-terminal end of the intracellular domain using a 9 amino acid flexible linker; **B-D** , nuclear localization of LIN-12::mNG::3xFlag in specific cells correlates with known Notch signaling events in *C. elegans* larval development. Arrowheads indicate cells with nuclear signal; **B** , LIN-12::mNG::3xFlag shows nuclear localization in ventral, but not dorsal, M lineage progenitors; **C** , LIN-12::mNG::3xFlag localization in VPCs is consistent with genetically determined functions in VPC specification. LIN-12::mNG::3xFlag exhibits clear nuclear localization in P5.p and P7.p cells where Notch signaling is known to be active, but localization is restricted to cell membrane and cytoplasmic domains in the neighboring P4.p, P6.p, and P8.p cells where Notch signaling is not active at this time; **D** , LIN-12::mNG::3xFlag nuclear localization in presumptive spermatheca (left) and central somatic gonad precursors (center); **E** , time-lapse imaging of LIN-12 intracellular domain localization in a dividing P7.p cell and its daughters reveals dynamic Notch activity in real time. Asterisk at 30 minutes indicates P7.p division. Nuclear LIN-12::mNG::3xFlag is apparent in P7.p prior to division and in both daughter cells immediately afterwards. Nuclear signal diminished over time in the posterior daughter P7.pp and was maintained in the anterior daughter P7.pa. Nuclei are outlined at key time points (0, 35, 85 minutes), and time elapsed is displayed as minutes:seconds; **F-I** , K-NAA treatment can be used to deplete endogenously tagged LIN-12::mNG::3xFlag::AID *in vivo* ; **F** , nuclear LIN-12::mNG::3xFlag::AID fluorescence is visible in P5.p, P7.p, and several somatic gonad cells in a control animal imaged with moderate laser power; **G** , mCherry::HIS-11 fluorescence marks nuclei of cells that express TIR1; **H** , merged image showing LIN-12::mNG::3xFlag::AID, mCherry::HIS-11, and DIC. Arrowheads indicate cells with visible nuclear NICD signal; **I** , even with maximal laser power, LIN-12::mNG::3xFlag::AID fluorescence was barely visible in a representative animal treated with the synthetic auxin analog K-NAA. The bright circular punctae are due to autofluoresence in intestinal cells that is more apparent with higher excitation intensity; **J** , mCherry::HIS-11 marks nuclei of cells that express TIR1; **K** , merged image showing LIN-12::mNG::3xFlag::AID, mCherry::HIS-11, and DIC. Nuclear NICD signal was not observed in any cells. All images are oriented with anterior to left and dorsal to top. Scale bars = 10 um.

## Description

Cell-cell communication through evolutionarily conserved signaling pathways orchestrates many aspects of animal development and homeostasis. To understand signaling mechanisms at the cellular level, it is important to develop tools to visualize endogenous signaling pathway activity and to manipulate signaling in time and space. Notch signaling is one widely conserved cell-cell signaling pathway that mediates cellular interactions in numerous contexts. Receptors in the Notch family play important roles in key developmental events including cell fate specification, differentiation, proliferation, apoptosis, migration, and morphogenesis (reviewed in Artavanis-Tsakonas *et al* ., 1999, and Hori *et al.* , 2013). Understanding mechanisms of Notch signaling is also clinically relevant as Notch signaling has been implicated in multiple human diseases (reviewed in Radtke & Raj, 2003, and Siebel & Lendahl, 2017).

Among metazoan cell-cell signaling pathways, the mechanism of Notch signal transduction is startlingly direct. The Notch protein is a transmembrane receptor that, upon activation by its extracellular ligand, undergoes proteolytic cleavages that release the Notch intracellular domain (NICD) from the cell membrane. The NICD then translocates into the nucleus where it acts as part of a transcriptional complex to regulate expression of target genes (Struhl *et al* ., 1993; Struhl & Adachi, 1998). *C. elegans* has been used for decades as a tractable and powerful model to elucidate mechanisms of Notch signaling *in vivo* (reviewed by Greenwald, 2005; Greenwald & Kovall, 2013). The *C. elegans* genome encodes two Notch proteins, LIN-12 and GLP-1, that have largely non-overlapping functions. During development, GLP-1 functions primarily in the germline and early embryo, and LIN-12 regulates multiple cell interactions and fate decisions in embryonic and larval somatic tissues (reviewed by Greenwald, 2005; Kimble & Crittenden, 2005; Priess, 2005).

Researchers have developed several methods to visualize dynamic Notch activity in *C. elegans* using live imaging of Notch target gene expression (Kershner *et al.* , 2014; Lee *et al.,* 2019) or changes in biosensor localization (Shaffer & Greenwald, 2022). Because nuclear translocation of the NICD is directly related to its activity, it is logical that live imaging of an endogenously tagged NICD could also provide a readout for Notch signaling in real-time. Such an approach has been demonstrated with live imaging of GLP-1::sfGFP and GLP-1::HaloTag transgenes (Sorensen *et al.,* 2020), but has been thought to be impractical for endogenous LIN-12 based on low levels of endogenous expression in key cell types combined with high protein turnover rates (Attner *et al.,* 2019; Shaffer & Greenwald, 2022). Here, we report a novel allele of *lin-12* endogenously tagged with the rapidly maturing, bright green/yellow fluorescent protein mNeonGreen (mNG) (Shaner *et al.,* 2013), which we found made it possible to visualize dynamic nuclear localization of the endogenous LIN-12 intracellular domain *in vivo* using spinning disk confocal microscopy. We also report an endogenously tagged *lin-12::mNG::3xFlag::AID* allele that made it possible to conditionally degrade endogenous LIN-12 protein using well characterized auxin-inducible degradation methods (Zhang *et al* ., 2015; Martinez *et al* ., 2020). This toolkit complements existing methods and provides a direct approach to investigate LIN-12/Notch signaling mechanisms in *C. elegans* .

To visualize LIN-12 intracellular domain localization *in vivo* , we used CRISPR/Cas9-triggered homologous recombination (Dickinson *et. al.,* 2013; Dickinson *et al.,* 2015) to insert mNG::3xFlag at the carboxy-terminus of endogenous *lin-12* (Fig. 1A). Homozygous *lin-12::mNG::3xFlag* animals were phenotypically indistinguishable from wild-type, lacking *lin-12* loss of function phenotypes. We conclude this knock-in allele encodes a biologically functional Notch protein. Spinning disk confocal microscopy revealed that tagged LIN-12 was expressed in patterns generally consistent with its known functions. As expected, LIN-12::mNG::3xFlag localized primarily to the cell membrane, along with localized cytoplasmic domains that may represent endosomes. Strikingly, we also observed clear nuclear localization of the endogenously tagged NICD in cells where and when LIN-12 has well-documented functions (Fig. 1B-E).

Focusing on larval development, we found that LIN-12::mNG::3xFlag was expressed in all postembryonic mesoderm progenitor cells at the 4M and 8M stages, but was only localized to the nucleus in ventral M-lineage cells (Fig. 1B), consistent with functions in dorsoventral patterning of the M lineage (Foehr & Liu, 2008). LIN-12::mNG::3xFlag was subsequently visible in somatic gonad cells and in several PN.p cells (Fig. 1C) where *lin-12* regulates cell fates (reviewed by Sternberg, 2005). LIN-12::mNG::3xFlag was visible at the cell membrane and in internalized punctae in the P3.p – P8.p cells but localized to the nucleus only in P5.p and P7.p (Fig. 1C). NICD nuclear localization specifically in these cells is consistent with functions for *lin-12* in specifying 2 ^o^ vulval precursor cells (reviewed by Sternberg, 2005). An accompanying Micropublication by Medwig-Kinney *et al* . describes LIN-12 dynamics during AC/VU specification and presents additional tools for visualizing Notch/Delta feedback in these cells (Medwig-Kinney *et al.,* in press).

Following vulval precursor cell patterning, LIN-12::mNG::3xFlag was highly expressed in numerous somatic gonad cells, with nuclear localization in several spermatheca and uterine precursor cells (Fig. 1D). To assess the utility of endogenously tagged LIN-12 for visualizing changes in Notch activity over time, we examined time-lapse images of LIN-12::mNG::3xFlag localization in the P7.p cell and its descendants over 90 minutes during the process of VPC specification. Nuclear signal was prominent in P7.p, diminished during the process of cell division, and initially reappeared in both daughter cells. Nuclear signal subsequently disappeared in the posterior daughter P7.pp but was maintained in the anterior daughter P7.pa (Fig. 1E), which remains in contact with ligand-expressing P6.p descendants. Although we observed transient nuclear NICD localization in both daughter cells immediately after P7.p cell division, it is unclear whether LIN-12 has developmental functions in P7.pp after its birth. One possibility is that cleaved NICD may be present in the cytoplasm during P7.p division, is inherited equally, and translocates into the nuclei of both daughter cells after division is complete. Subsequent loss of nuclear signal in P7.pp could represent normal protein turnover. NICD localization exhibited similar dynamics in P5.p (not shown). These observations suggest that endogenous NICD localization can be used as a direct readout of LIN-12 signaling *in vivo* .

Thoroughly understanding how signaling pathways function requires the ability to manipulate signaling in space and time. To make progress towards this goal, we generated an additional strain where *lin-12* is endogenously tagged with mNG::3xFlag and an auxin-inducible degron (AID). The AID system allows for conditional degradation of degron-tagged proteins in cells that express TIR1, which mediates proteasomal degradation of AID-tagged targets upon treatment with auxin (Zhang *et al* ., 2015; Martinez *et al* ., 2020). As a proof of principle experiment, we combined *lin-12::mNG::3xFlag::AID* with an existing strain that ubiquitously expresses *TIR1::T2A::mCherry::his-11* and tested for the ability to degrade LIN-12 protein (Hills-Muckey *et al.,* 2022). In control animals treated with water, LIN-12::mNG::3xFlag::AID expression and subcellular localization patterns appeared indistinguishable from LIN-12::mNG::3xFlag (Fig. 1F-H). Following treatment with the synthetic auxin K-NAA (1 mM in solid media), we observed a strong reduction of LIN-12::mNG::3xFlag::AID fluorescence (Fig. 1I-K), which indicates this approach is a promising tool to conditionally target LIN-12 signaling. We did not observe uterine or vulval cell phenotypes in the absence of auxin, although we recognize that in other contexts Notch signaling may be more sensitive to auxin-independent degradation that has been observed with wild-type *_At_* TIR1 (Martinez *et al* ., 2020). Therefore, we also generated strains with a mutant version of the TIR1 transgene ( *_At_* TIR1(F79G)) that improves specificity (Hills-Muckey *et al* ., 2022). Upon treatment with a modified auxin (5-Ph-IAA), these strains exhibited phenotypes at comparable penetrance (not shown). Additional TIR1 and TIR1(F79G) lines available through the *Caenorhabditis* Genetics Center, or generated by users, could be used to leave the red channel open for visualization of other proteins and/or for tissue-specific degradation (Ashley *et al.,* 2021; Negishi *et al.* , 2022).

In summary, we expand the toolkit for investigating Notch signaling in *C. elegans* by providing new strains to visualize and conditionally perturb LIN-12/Notch signaling *in vivo* . Given the evolutionarily conserved nature of Notch signal transduction, we expect that similar approaches may be feasible in other animals amenable to live imaging. In comparison to previous methods for visualizing Notch activity in living *C. elegans,* live imaging of NICD localization has advantages and disadvantages that should be considered in the context of specific experimental designs and goals. A unique advantage is that visualizing the NICD itself allows researchers to explore additional questions related to how Notch proteins are localized within cells, how endocytosis is regulated (Shaye & Greenwald, 2002), and the potential for non-canonical Notch signaling processes that do not rely on nuclear translocation of the NICD (Sieiro *et al* , 2016; Polacheck *et al.,* 2017). Compared to a complementary red/green biosensor-based approach for visualizing LIN-12 activity (Schaffer & Greenwald, 2022), our method requires imaging only a single tagged protein and allows researchers to utilize red fluorescent proteins for other purposes. However, SALSA may be the preferred method for visualizing LIN-12 activity in cases where automated nuclear segmentation is desired or where membrane and/or endosome-localized LIN-12::mNG interferes with imaging nuclear localization. It is our hope that these new reagents will be useful to the *C. elegans* community to further study mechanisms of Notch signaling and its functions during development and homeostasis.

## Methods

**Strain maintenance**

*C. elegans* were reared on NGM plates under standard conditions and cultured at 20 ^o^ C. Strains generated in this study can be found in the Strain Table and will be deposited at the *Caenorhabditis* Genetics Center for use by the *C. elegans* community.

**Genome engineering**

mNG::3xFlag and mNG::3xFlag::AID were inserted at the carboxy-terminus of endogenous *lin-12* locus using CRISPR/Cas9-triggered homologous recombination with the self-excising selection cassette (SEC) method (Dickinson *et al.,* 2015). The guide RNA targeting sequence 5’- GGTTCGGAGTATCGCGTCAT-3’ was inserted into pDD162 (Peft-3>Cas9 + empty sgRNA, Addgene plasmid #47549) using Q5 site-directed mutagenesis (New England Biolabs). Homologous repair templates were generated by cloning PCR-amplified genomic homology arms into the vector backbones pDD268 (mNG^SEC^3xFlag, Addgene plasmid #132523) or pUA77 (mNG^SEC^3xFlag::AID) as described in detail previously (Dickinson *et al.,* 2015).

To generate knock-in strains, young adult worms were microinjected with an injection mix consisting of 50 ng/ml sgRNA + Cas9 plasmid, 10 ng/ml homologous repair template, and a co-injection mix of fluorescent array markers (Dickinson *et al.,* 2015). Knock-in animals were selected using hygromycin-B resistance, a roller phenotype, and lack of extrachromosomal arrays derived from the co-injection markers as described in detail previously (Dickinson *et al.,* 2015). After isolating homozygous roller strains, L1 animals were heat shocked at 34 ^o^ C for four hours to excise the SEC. Genome edits were confirmed by visualization of fluorescence and genotyping.

**Auxin-inducible degradation:**

Nematode growth media plates were treated with naphthaleneacetic acid (K-NAA) (PhytoTechnology Laboratories) diluted in water to provide a final concentration of ~1 mM K-NAA in solid media (Martinez & Matus, 2020). To degrade LIN-12::mNG::3xFlag::AID, L1 stage animals were moved to treated plates seeded with OP50 *E. coli* . Control plates were treated with an equivalent volume of water. The orthogonal auxin analog, 5-Ph-IAA, should be paired with the *_At_* TIR1(F79G) transgenes (Hills-Muckey *et al.* , 2022).

**Live imaging**

Imaging was performed using a Yokogawa CSU-W1 SoRa spinning disk confocal and Hamamatsu ORCA Fusion camera mounted on a Nikon Ti2 inverted microscope. We utilized 514 nm laser excitation and a 545/40 emission filter to image mNG in order to minimize autofluorescence that is prominent with 488 nm excitation (Heppert *et al.* , 2016). For imaging, we utilized the SoRa disk with either a 60x 1.27 NA water immersion objective and 2.8x SoRa magnifier or a 100x 1.49 NA oil immersion objective and 1.0x SoRa magnifier. Images were acquired using Nikon NIS Elements 5.3 software. For imaging, animals were anesthetized with 0.1 mmol/L levamisole in M9 buffer or immobilized using 0.1 mm polystyrene beads (Polysciences) (Kim *et al.,* 2013) and mounted on 4% agarose pads.

**Image processing**

Image brightness and contrast were adjusted using Fiji/ImageJ (v2.3.0) (Schindelin *et al.,* 2012). Figure was prepared using Adobe Illustrator (v24.1).

## Reagents

**Strain table**

**Table:** 

**Strain**	**Genotype**	**Source**
APL31	*lin-12* ( *ljf31* [ *lin-12* ::mNeonGreen[C1]^loxP^3xFLAG]) III.	This study
DQM1070	*cshIs128* [P *rpl-28* >TIR1::T2A::mCherry::his-11)] II; *lin-12* ( *ljf33* [ *lin-12* ::mNeonGreen[C1]^loxP^3xFLAG::AID]) III; *lag-2* ( *bmd202* [ *lag-2* ::P2A::H2B::mTurquoise2^lox511^2xHA]) V;.	This study
DQM1072	*cshIs140* [P *rpl-28>* TIR1(F79G)::T2A::mCherry::his-11] II; *lin-12* ( *ljf33* [ *lin-12* ::mNeonGreen[C1]^loxP^3xFLAG::AID]) III; *lag-2* ( *bmd202* [ *lag-2* ::P2A::H2B::mTurquoise2^lox511^2xHA]) V.	This study

**Plasmids**

**Table:** 

**Plasmid**	**Description**	**Source**
pAP163	*Peft-3>Cas9 + PU6>lin-12* sgRNA plasmid	This study
pAP162	*lin-12* mNeonGreen[C1]^SEC^3xFLAG repair plasmid	This study
pTG024	*lin-12* mNeonGreen[C1]^SEC^3xFLAG::AID repair plasmid	This study
